# Technique of Intravenous Regional Block for Complex Regional Pain Syndrome Using Methylprednisolone, Lidocaine, and Labetalol

**DOI:** 10.7759/cureus.118301

**Published:** 2026-10-21

**Authors:** Parker L Dillin

**Affiliations:** 1 Orthopedic Surgery, Texas Health Resources, Fort Worth, USA

**Keywords:** bier block, complex regional pain syndrome, intravenous regional block, labetalol, lidocaine, methylprednisolone, neuromodulation, pain management

## Abstract

Intravenous regional block (IVRB), adapted from the Bier block technique, is a minimally invasive, low-risk interventional option for complex regional pain syndrome (CRPS). This technical report describes a reproducible IVRB protocol using methylprednisolone, lidocaine, and labetalol and emphasizes procedural details that may influence treatment delivery. Key elements include obtaining venous access distal to the involved region, selecting tourniquet position and pressure according to the affected extremity, slowly infusing the injectate, and performing deep friction massage while the extremity remains isolated from the systemic circulation. The report also describes staged tourniquet deflation, the use of anesthesia to limit procedure-related pain, an initial series of repeated blocks before judging response, and subsequent adjustment of block frequency according to the duration of pain relief. Practical methods for obtaining venous access in an edematous hand or foot and approaches to patients with remission, palliation, or an inadequate response are discussed. The objective is to provide clinicians with a detailed technique that can be reproduced and critically evaluated in the treatment of CRPS.

## Introduction

Intravenous regional block (IVRB) for complex regional pain syndrome (CRPS) is an adaptation of the Bier block technique. In patients with CRPS, the goal of IVRB is to desensitize involved areas with a combination of nerve membrane-stabilizing drugs and massage. This technical report describes the author’s IVRB protocol using methylprednisolone, lidocaine, and labetalol, with emphasis on procedural details intended to improve consistent delivery of the injectate and minimize avoidable causes of technical failure.

## Technical report

The patient is brought to the treatment area. After administration of anesthesia, a tourniquet is applied above the site of CRPS (the site): the thigh for knee CRPS, the calf for foot and ankle CRPS, the brachium for elbow involvement, and the forearm for the condition in the hand and wrist. The tourniquet pressure is initially set to approximately 20 mm below systolic pressure, and the involved area is placed in a dependent position to induce venous distention. It is important to insert a 23-gauge butterfly into a vein distal to the site. Importantly, in the sole report finding no benefit from lidocaine-steroid IVRBs, the investigators were unable to obtain venous access distal to the involved site because swelling prevented venous access in the hand [1]. This technical difference may have materially affected delivery of the injectate to the involved tissues and therefore the efficacy of the procedure. In this author’s experience in performing over 3,000 IVRBs, difficulty in achieving venous access in the foot or hand can always be resolved by using the following methods. First, venous distention increases the longer the extremity is dependent; in some cases, dependence of up to 15 minutes is necessary to visibly distend veins. Second, very warm packs wrapped around the intended access point increase venous visibility, especially if replaced every few minutes before they cool. Third, application of topical 2% nitroglycerin ointment at the intended site of venipuncture is helpful. Finally, a “milking” technique uncovers veins hidden by edema: manual pressure is applied and held over the suspected site of veins until all edema is expressed from the area, unveiling the previously hidden veins.

After establishing venous access, tourniquet pressure is set to 400 mmHg for thigh tourniquets, 350 mm for calf tourniquets, and 300 mm for brachial or forearm tourniquets. The proceduralist maintains the position of the butterfly while an assistant slowly infuses the injectate. For IVRBs in adult patients, 125 mg of methylprednisolone, 4mg/kg of lidocaine without epinephrine and 0.4mg/kg of labetalol are used. For all upper extremity procedures, and lower extremity procedures using a calf tourniquet 0.5% lidocaine is used. For lower extremity procedures requiring a thigh tourniquet, 0.25% lidocaine is used. For patients requiring long-term block repetition for palliation, the steroid is omitted from the injectate or is used intermittently.

After completing the intravenous infusion, the access is removed, and deep friction massage is performed at the site for 20 minutes with CBD cream.

At 20 minutes after initiation of massage, the tourniquet is deflated for five seconds, permitting a minimal amount of the injectate that has not been tissue-bound to enter the venous circulation. The tourniquet is re-inflated for five minutes, and massage is continued. The tourniquet is deflated a second time for five seconds, then re-inflated for five minutes with continued massage. The tourniquet is then permanently deflated, and the patient is taken to the recovery room.

Both the use of a tourniquet and the deep friction massage during IVRB augment pain. Anesthesia is necessary because of the pain caused by the tourniquet, deep friction massage of hypersensitive tissues, and mobilization/manipulation of painful joints. In the author’s experience, use of sedation is insufficient and general anesthesia is preferable for several reasons. First, pain caused by the procedure can be more effectively prevented using general anesthesia than sedation. Additionally, there is a clear value in minimizing the pain inflicted on the patient from this procedure. Second, most patients with CRPS are already miserable due to chronic, unrelenting pain. Prevention of pain caused by the procedure with general anesthesia enhances patient compliance with block repetitions sufficient to completely suppress their pain or achieve reasonable palliation. Third, anything that increases the burden of pain patients with CRPS experience may fuel and sustain the syndrome. For instance, patients have often complained to the author that their condition has been worsened by pain experienced due to aggressive physical therapy. Similarly, patients often report that other painful events, such as minor or major trauma to the involved area, result in a fundamental worsening of their CRPS. The fact that the pain of CRPS is by definition disproportionate to the cause highlights these patients’ increased sensitivity to painful stimuli. These facts argue for minimizing the “pain burden” patients with CRPS experience. General anesthesia minimizes the pain burden of IVRB. Technical pearls for performing IVRB are summarized in Table 1.

**Table 1:** Technical pearls for intravenous regional block.

Pearl	Application in the author’s protocol
Obtain access distal to the involved region	Place a 23-gauge butterfly in a vein distal to the affected site to deliver the injectate to the involved tissues
Allow time for venous distention	Keep the extremity dependent with initial tourniquet pressure approximately 20 mmHg below systolic pressure. In difficult cases, allow up to 15 minutes for veins to become visible
Improve venous visibility	Apply warm packs around the intended access point, replacing them before they cool. Topical 2% nitroglycerin ointment at the intended venipuncture site may also help
Displace edema hiding veins	Apply and hold manual pressure over the suspected vein until edema is expressed from the area and the vein becomes visible
Stabilize access during infusion	Have the proceduralist maintain the butterfly position while an assistant slowly infuses the injectate
Continue massage during staged release	After the initial 20 minutes of deep friction massage, continue massage through two cycles of five-second tourniquet deflation followed by five-minute reinflation before final release
Minimize procedural pain	The author prefers general anesthesia to prevent pain from the tourniquet, deep friction massage, and joint mobilization or manipulation, and to support adherence to repeated blocks

A series of three to five weekly blocks is performed until the patient’s response can be assessed. The most common response to the initial series of IVRBs is a mild increment in pain after the first block, an equivocal response after the second, and improvement that incrementally increases after the third to fifth blocks. Repetition of IVRBs after the first series is based on the patient’s response to the blocks, which can be categorized as (complete pain) remission, palliation, or ineffectiveness. The term “remission” is used in this paper in preference to “cure” because recurrence of CRPS in cases that had previously been thought to be cured has been documented [2-7].

For patients for whom complete remission of pain is eventually achieved, weekly blocks are continued until complete pain relief is achieved for the entire week following the block, at which time block frequency is reduced to every other week. If pain relief is maintained for the full two weeks after blocks, the frequency is tapered to every three and then four weeks and then discontinued. Some patients progress very rapidly through this tapering frequency. If symptoms recur during tapering, the frequency of blocks is adjusted until pain relief is durable between blocks, at which time an effort to taper is made again. The number of blocks to achieve complete remission has ranged from a single block (rarely) to 30 or more blocks. In patients who are making incremental improvement with IVRBs, the proceduralist should not be deterred by the number of blocks it is taking to achieve that progress, bearing in mind that complete remission of long-standing CRPS has not been reliably achieved with any other combination of methods. Further, remission means avoidance of insertion of spinal cord or dorsal root ganglion stimulator, both of which require invasion of uninvolved core anatomy.

When patients cannot be weaned from blocks due to pain recurrences between blocks, several options are available to the patient. First, blocks can be continued at a frequency that the patient chooses and adjusts according to their pain needs. The value of prolonged repetitive blocks has previously been reported in a patient who did not want a stimulator and whose pain was controlled by 158 blocks during a seven-year period [8].

For the rare patient who receives comparable relief from sympathetic blocks, these can be substituted for IVRBs or a protocol devised to alternate between sympathetic blocks and IVRBs. However, the vast majority of patients report such superior pain relief from IVRBs that they do not want further sympathetic blocks.

When patients experience severe pain that is not suppressed by IVRBs, a trial of spinal cord or dorsal root ganglion stimulation may convince patients to elect for a permanent stimulator. However, in this author’s experience, it is common for patients undergoing permanent stimulator insertion to experience relief of only about 50% of their pain, or initial improvement that fades over time or during periods of stimulator dysfunction. The author’s experience mirrors the reported results of spinal cord stimulation for CRPS, which have been repeatedly reported as successful when at least 50% pain relief is achieved [9-14]. For these patients, IVRBs can add to the quality of pain alleviation and quality of life provided by the stimulator.

## Discussion

IVRBs using methylprednisolone, lidocaine, and labetalol represent a tool in the treatment of CRPS that pain management physicians rarely use. The effective employment of IVRBs, in addition to other more widely used treatment methods, can result in complete remission of CRPS in many cases, and profound palliation of pain in many others [8,15-19]. As it has been advocated that instrumentation of the spinal vault be considered only when less invasive methods have failed [20-25], IVRBs provide the pain management physician a tool that can avert the need for or forestall the timing of spinal cord or dorsal root ganglion stimulation.

Repetitive intravenous regional blocks versus sympathetic block

Most patients prefer and are less apprehensive of IVRBs than having a needle inserted in their neck or back for sympathetic blocks. It is rare in the author’s experience for a patient to report comparable pain relief from sympathetic blocks.

Adequacy of series

A trial of only one or two IVRBs is insufficient to determine the value of these blocks for the vast majority of patients, particularly those with long-standing CRPS. A series of three, or preferably five, blocks is necessary to fully allow patients to experience the benefits of IVRB. These patients are commonly depressed and demoralized from the unrelenting and severe pain they have experienced over prolonged periods. Many patients with CRPS have gone years before an accurate diagnosis, have been misdiagnosed or labeled as hypochondriacs or malingerers by multiple doctors, or have experienced little or no pain relief from other methods. Many report an increase in pain from physical therapy, which they have endured in the hope of improvement. Many do not like the side effects and inadequacy of pain relief from prescribed gabapentin and pregabalin. Their adherence to a block regimen is often challenged by the inadequacy of pain relief from the initial or second block. It is very important to set patient expectations before initiating the initial series of IVRBs. They should be warned not to judge the effectiveness of the blocks until the series is complete.

The pattern of pain patients experience with the initial series of blocks is variable. For instance, some patients even with severe and long-standing symptoms experience dramatic pain relief from the first block. This is usually a favorable predictor for achieving complete remission of pain with IVRBs. Occasionally, remission of pain is achieved with a single block.

Dealing with pain worsening after the first block

Rarely, patients will experience a dramatic worsening of pain following the first block. Monitoring these patients for another month or two may uncover that some experience noticeable pain relief in the weeks after. While the initial patient response to a significant increase in pain is to discontinue the series, patients who experience delayed benefit may opt for a repeat trial of IVRBs. If the experience following the second block is the same as the first, it is probably wisest to abandon this method in the treatment of their CRPS. However, if resumption of blocks is followed by pain improvement, then it is reasonable to proceed with this paper’s protocol.

Between intravenous regional blocks

Finally, it is worth revisiting the concept of minimizing pain burden in CRPS patients. While repetitive Cochrane Database Systematic Reviews and other reviews have identified that the evidence that physical therapy is beneficial in CRPS is low quality [26-29], this author has found that patients report a pain benefit in at least daily self-administered massage of the area affected by CRPS using CBD cream in the intervals between blocks. Pearls for assessing response and adjusting repeat blocks are summarized in Table 2.

**Table 2:** Pearls for assessing response and adjusting repeat blocks.

Pearl	Application in the author’s protocol
Set expectations before starting	Explain that benefit may be delayed. The author generally uses three to five weekly blocks before assessing response; an equivocal response to the first or second block need not indicate failure
Reassess marked initial worsening	If pain worsens dramatically after the first block, monitor for delayed benefit over one to two months. A repeat trial may be considered if delayed benefit occurs; similar worsening after the second block favors abandoning the method
Taper according to the duration of relief	Continue weekly blocks until complete pain relief lasts the entire week. Extend intervals to two, three, and then four weeks as relief remains durable, then discontinue
Adjust when symptoms recur	If pain returns during tapering, adjust block frequency until relief again lasts between blocks, then retry tapering. For ongoing palliation, individualize frequency according to the patient’s pain needs
Modify steroid use during long-term palliation	When prolonged block repetition is required for palliation, omit methylprednisolone or use it intermittently
Support comfort between blocks	Encourage at least daily self-administered massage of the affected area using cannabidiol cream, as described in the author’s practice

## Conclusions

IVRBs using methylprednisolone, lidocaine, and labetalol provide an additional interventional option for selected patients with CRPS. When effectively incorporated with other treatment methods, IVRBs may provide substantial palliation and, in some patients, durable remission of pain. Consistent with reserving instrumentation of the spinal canal for patients in whom less invasive methods have failed, IVRBs may avert the need for, or forestall the timing of, spinal cord or dorsal root ganglion stimulation.
