# Correction: Technique of Intravenous Regional Block for Complex Regional Pain Syndrome Using Methylprednisolone, Lidocaine, and Labetalol

**DOI:** 10.7759/cureus.c499

**Published:** 2026-11-02

**Authors:** Parker L Dillin

**Affiliations:** 1 Orthopedic Surgery, Texas Health Resources, Fort Worth, USA

Dosages in paragraph 2 of the Technical Report section have been corrected as follows: “For IVRBs in adult patients, 125 mg of methylprednisolone, 4mg/kg of lidocaine without epinephrine and 0.4mg/kg of labetalol are used. For all upper extremity procedures, and lower extremity procedures using a calf tourniquet 0.5% lidocaine is used. For lower extremity procedures requiring a thigh tourniquet, 0.25% lidocaine is used.”

